# Evidence for natural recombination between mink enteritis virus and canine parvovirus

**DOI:** 10.1186/1743-422X-9-252

**Published:** 2012-10-30

**Authors:** Jianke Wang, Shipeng Cheng, Li Yi, Yuening Cheng, Shen Yang, Hongli Xu, Hang Zhao, Xijun Yan, Hua Wu

**Affiliations:** 1 State Key Laboratory for Molecular Biology of Special Economic Animals, Institute of Special Economic Animal and Plant Sciences, Chinese Academy of Agricultural Sciences, 4899 Juye Street, Changchun, 130112, China

**Keywords:** Parvovirus, Mink enteritis virus, Recombination

## Abstract

A virus was isolated from mink showing clinical and pathological signs of enteritis in China. This virus, designated MEV/LN-10, was identified as mink enteritis virus (MEV) based on its cytopathic effect in the feline F81 cell line, the hemagglutination (HA) and hemagglutination inhibition (HI) assay, electron microscopy (EM) and animal infection experiments. The complete viral genome was cloned and sequenced. Phylogenetic and recombination analyses on the complete MEV/LN-10 genome showed evidence of recombination between MEV and canine parvovirus (CPV). The genome was composed of the NS1 gene originating from CPV while the VP1 gene was of MEV origin. This is the first demonstration of recombination between a CPV and MEV in nature. Our findings not only provide valuable evidence indicating that recombination is an important genetic mechanism contributing to the variation and evolution of MEV, but also that heterogeneous recombination can occur in the feline parvovirus subspecies.

## Background

Mink enteritis virus (MEV) is an autonomous parvovirus causing an acute disease in mink [1]. The disease was initially reported by Schofield in 1949 in Canada [2] while the isolation and identification of the viral pathogen did not occur until 1952 [3]. Since then, the disease has been reported in a number of other countries, and now is thought to occur wherever mink are farmed [4]. MEV belongs to the genus Parvovirus within the family Parvoviridae and has been included in the feline parvovirus subspecies together with feline panleukopenia virus (FPLV), canine parvovirus (CPV) and raccoon parvovirus (RPV) [5]. These viruses are highly homologous under field and laboratory conditions and share many antigenic features [6]. Feline parvovirus subspecies are autonomous, single-stranded DNA viruses that have a genome length of approximately 5,000 bp [4,7,8]. The genome encodes for two nonstructural proteins, NS1 and NS2, and two capsid proteins VP1 and VP2.

Genetic recombination is generally considered a key mechanism in virus evolution. Recently, a Japanese research group provided the first clear evidence of recombination both between different CPV antigenic types and between CPV and FPLV under field conditions [9,10]. With this in mind, we undertook phylogenetic and recombinant analyses on the complete genome of an MEV isolated from minks in China. Our results indicate that the isolate was most likely generated by recombination between CPV and MEV. This is the first report of such a recombination between CPV and MEV in nature.

## Methods

### MEV isolation

An MEV strain was recovered from clinically diseased minks. In August 2010, a severe outbreak of haemorrhagic enteritis occurred in a farm in Dandong City, Liaoning province, China consisting of approximately 400 minks (*Mustela vison*) raised for fur production. Approximately 200 (50%) of the minks suffered from haemorrhagic diarrhea of which 25% (100) died within 7-10 days after the onset of clinical signs. The intestinal contents were collected from diarrheic minks, and the virus was isolated as described elsewhere [11]. Briefly, the faecal samples were homogenized in PBS and subsequently clarified by centrifugation. The samples were treated with antibiotics and were then inoculated into F81 cells [12]. Inoculated cell cultures showing cytopathic effects were used for DNA extraction, in addition to morphological, serological, physical and chemical property investigations using standard techniques [12,13].

### DNA extraction and PCR

The full-length genome of the virus was sequenced according to methods described previously [14]. Four overlapping fragments covering the whole viral genome were amplified by PCR using the corresponding primers. Briefly, the cell cultures were boiled for 10 min and centrifuged at 9,000 *g* for 2 min. The resulting supernatant was used as the template for PCR amplification, in a final volume of 25 μl containing 2.5 μl of 10 X LA Taq buffer, 2 μl of dNTPs (2.5 mM), 10 pmol each of the corresponding primers, 0.5 μl LA Taq polymerase (2.5 U/ml) (TaKaRa, Dalian, China) and 18 μl distilled H_2_O. The following thermocycling conditions for the PCR amplification were applied: 95°C for 5 min, followed by 35 cycles of denaturation at 94°C for 45 s, primer annealing at 55°C for 45 s, and extension at 72°C for 2 min, with a final elongation at 72°C for 8 min. The amplicons were analyzed by electrophoresis on a 1% agarose gel in Tris acetate EDTA buffer.

### Cloning and sequencing

PCR products of the correct size were gel purified and cloned into the pMD18-T vector (TaKaRa, Dalian, China). For each amplified genomic fragment, 3-5 positive recombinant plasmids were sequenced in both directions using the M13 primer (Shanghai Invitrogen Biotechnology Company, Shanghai, China) and a gene specific primer. The complete sequence determined in this study has been deposited with GenBank, under accession number HQ694567.

### Sequence analysis

Complete CPV, FPLV and MEV genomic sequences were obtained from GenBank (Table 1). These CPV, FPLV and MEV genomic sequences and the complete genome sequence of the MEV/LN-10 strain were aligned and analyzed using the ClustalW multiple alignment algorithm in the MegAlign program of the DNASTAR software suite. The putative recombinant sequence and its parents were identified using two different recombination detection programs i.e. Genetic Algorithms for Recombination Detection (GARD) and the RDP3 software package [15-22]. General parameter settings were used for GARD. The RDP3 program includes six separate recombination detection programs and enables automated analyses of nucleotide sequence alignment using all of the programs.

**Table 1:** Nucleotide sequence accession numbers of MEV, CPV and FPLV isolates analyzed in this study

**No.**	**Strains**	**Accession no.**	**Genetic type**	**Year submitted**	**Origin**
1	Abashiri	D00765	MEV	2007	Japan
2	MEVB	FJ592174	MEV	2009	China
3	**MEV/LN-10**	**HQ694567**	**MEV**	**2011**	**China**
4	CU-4	M38246	FPLV	1996	USA
5	193/70	X55115	FPLV	2005	USA
6	XJ-1	EF988660	FPLV	2007	China
7	FPV-8a.us.89	EU659113	FPLV	2008	USA
8	FPV-4.us.64	EU659112	FPLV	2008	USA
9	FPV-3.us.67	EU659111	FPLV	2008	USA
10	FPV-kai.us.06	EU659115	FPLV	2008	USA
11	FPV-8b.us.89	EU659114	FPLV	2008	USA
12	CPV-N	M19296	CPV-2	1995	USA
13	CPV-b	M38245	CPV-2	1996	USA
14	Y1	D26079	prototype CPV-2a	2002	Japan
15	CPV2a	AJ564427	new CPV-2a	2004	India
16	CPV-193	AY742932	new CPV-2b	2005	USA
17	CPV-339	AY742933	new CPV-2a	2005	New Zealand
18	CPV-447	AY742934	new CPV-2b	2005	USA
19	CPV-U6	AY742935	new CPV-2a	2005	Germany
20	CPV-395	AY742936	new CPV-2b	2005	USA
21	B-2004	EF011664	new CPV-2a	2006	China
22	CPV-13.us.81	EU659118	prototype CPV-2a	2008	USA
23	CPV-410.us.00	EU659119	new CPV-2b	2008	USA
24	CPV-411a.us.98	EU659120	new CPV-2b	2008	USA
25	CPV-411b.us.98	EU659121	new CPV-2b	2008	USA

### Phylogenetic analysis

The phylogenetic relationships between the different viral genomes were evaluated using MEGA version 5.0. Phylogenetic trees were constructed using the Maximum Likelihood method in MEGA5 and the Tamura-Nei model with 2000 bootstrap replicates to assess the confidence level of the branch pattern. Bootstrap values > 70% were considered to be significant. Nucleotide and amino acid sequences of MEV/LN-10 together with reference MEVs, and CPVs were analyzed using DNAMAN software.

## Results

### Virus isolation

Typical parvovirus associated CPE appeared in the F81 cells inoculated with the isolated virus. The negatively stained virus particles extracted from the cell supernatant following centrifugation were approximately 20 nm in diameter when examined by EM, and displayed a typical MEV morphology (data not shown). The virus was highly resistant to heat (56°C for 30 min), 20% ether, and acid (pH 3.0), but was susceptible to the antiviral agent 5-iododeoxyuridine (5-IUDR) (data not shown). The HA assay indicated that the highest HA titer of the virus was 1:2,048 with porcine erythrocytes but < 1:2 with sheep, guinea pig, rat, rabbit, chicken and human erythrocytes. The ability to agglutinate pig erythrocytes was inhibited by specific antiserum in the HI assay. In artificial infection experiments of mink, all of the animals injected subcutaneously with 1 × 10^5.5^ TCID_50_/ml of MEV/LN-10 presented clinical symptoms of disease consistent with parvovirus infection and the virus was subsequently recovered from the animals. All animal work and experimental procedures were approved by the Institutional Animal Care and Use Committee of Jinlin University, China.

### Analysis of viral sequences

**Figure 1:**
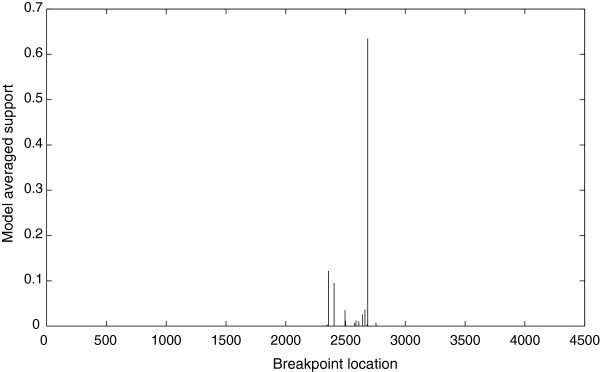
**Recombination breakpoint as identified by GARD.** The program identified nucleotide position 2,686 as a putative breakpoint for recombination.

**Figure 2:**
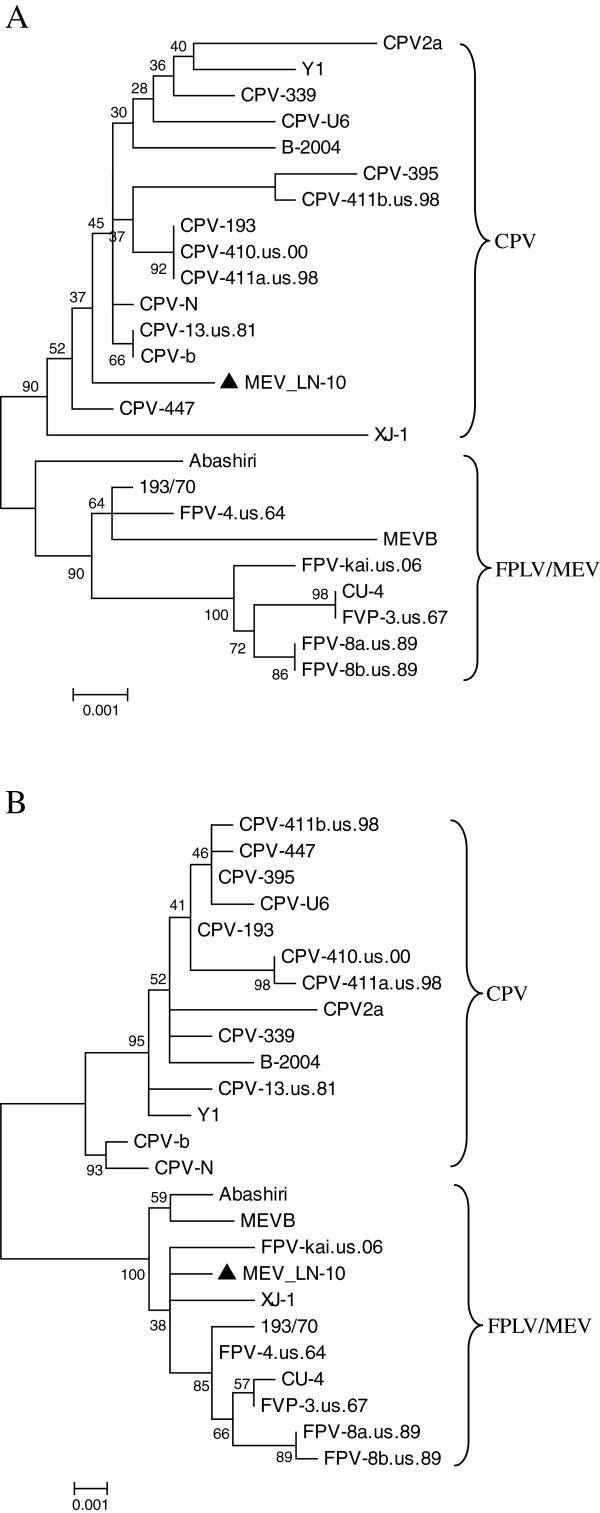
**Phylogenetic analysis of MEV/LN-10.** Nucleotide sequences (**A**) 1–2,685 and (**B**) 2,686–4,269 were separately analyzed using the Maximum Likelihood method and Tamura-Nei model in MEGA5. Groupings were tested by bootstrapping 2000 replicates and support percentages greater than 70% are marked at the corresponding nodes. Isolated MEV/LN-10 is marked by solid triangles.

Both the GARD and RDP3 programs detected a putative recombination breakpoint in the nucleotide sequence of MEV/LN-10. Apart from the recombinant FPLV strain XJ-1, there was no evidence supporting recombination events in the genome sequences of the remaining 23 strains of FPLV, MEV or CPV analyzed by GARD or RDP3. GARD identified nucleotide position 2,686 in the MEV/LN-10 as a putative breakpoint for recombination (Figure 1). The sequence was divided into two fragments at nucleotide position 2,686 and separate phylogenetic tress for each fragment were constructed (Figure 2). The first fragment (i.e. 1- 2,685) clearly clustered with the CPV clade (Figure 2A), while the second fragment (2,686–4,269) clustered within the FPLV/MEV clade (Figure 2B).

Moreover, a potential recombination event was detected using the MaxChi method in RDP3. MaxChi identified nucleotide position 2,707 as an ending breakpoint with a Bonferroni corrected *p*-value of < 0.05, and a predicted recombination region was located at around nucleotide positions 1,166 and 2,707 of the alignment. Among the nucleotide sequence data for all 24 strains entered into the RDP3 program, nucleotides 1,166-2,707 of the MEV/LN-10 possessed the greatest similarity (99.5%) to the corresponding part of strain CPV-447. The different breakpoint determined by MaxChi from that determined by GARD was due to MaxChi calculating the midpoint of nucleotide positions 2,680 and 2,752 as the putative breakpoint since no nucleotide difference was detected between these positions as determined from the alignments of MEV and CPV. The alignment was cut at two points (nucleotide positions 1,166 and 2,707) and separate phylogenetic trees for each of the three sections were constructed. Both the first (1–1,165) and the recombination (1,166–2,707) regions of the alignment clustered with the CPV clade while the third fragment (2,708–4,269) clustered with the FPLV/MEV clade. Analysis using other evolutionary models, such as the neighbor-joining method and Jukes–Cantor, produced trees with nearly identical topologies (data not shown).

### Sequence analysis of nucleotide and deduced amino acid sequences

A comparison of all the nucleotide variations and the deduced amino acid sequences of the NS1 and VP1 (VP2) proteins of the MEV/LN-10 strain with those of reference viruses MEVB and CPV-447 are presented in Table 2. The NS1 gene (1-2,685) shared nucleotide similarities with CPV-447 while the VP1 gene (2,753-4,269) was most similar to the VP1 gene of MEVB. Confirming this, examination of the NS1 protein (from the N-terminal to residue 595) revealed an amino acid sequence similar to CPV-447, while the VP1 protein (from residue 223 to the C-terminal) was similar to MEVB.

**Table 2:** Nucleotide sequence variations in the genes of MEV/LN-10 and amino acid variations in the NS1 and VP2 proteins of the MEV/LN-10 isolate

**nt position**	**28**	**288**	**741**	**743**	**1053**	**1107**	**1224**	**1251**	**1362**	**1401**	**1419**	**1576**	**1619**	**1633**	**1633**	**1686**	**1720**	**1785**	**1846**	**1887**	**1926**	**1959**
aa position of NS1	10	*	247	248	351	*	*	*	*	*	*	*	540	545	*	*	574	595	616	629	*	*
MEVB	I	c	H	T	N	a	t	a	t	c	g	c	V	Q	c	c	V	H	N	H	a	t
MEV/LN-10	V	t	Q	I	N	a	c	g	c	t	a	t	A	E	g	t	I	H	D	Q	g	c
CPV-447	V	t	Q	I	K	g	t	g	t	t	a	t	V	E	g	t	I	Q	D	Q	g	c
nt position	2076	2110	2355	2569	2586	2610	2640	2661	2753	2760	2773	2793	2816	2822	3051	3208	3213	3214	3403	3413	3427	3481
aa position of VP2	*	*	*	*	*	*	*	*	80	*	87	93	101	103	*	232	*	234	297	300	305	323
MEVB	g	c	a	c	a	t	a	g	K	a	M	K	I	V	t	I	t	H	S	V	D	D
MEV/LN-10	a	t	g	a	g	c	g	a	K	a	M	K	T	V	t	V	t	Y	S	V	D	D
CPV-447	g	t	g	a	g	t	g	g	R	g	L	N	T	A	c	I	c	H	A	G	Y	N
nt position	3552	3681	3746	3790	3918	4071	4137	4205	4217													
aa position of VP2	*	*	411	426	*	*	*	564	568													
MEVB	a	t	A	N	a	g	a	N	A													
MEV/LN-10	a	t	E	N	a	a	a	N	A													
CPV-447	g	c	E	D	g	a	g	S	G													

a) aa: amino acid, nt: nucleotide.

b) An asterisk (*) indicates a synonymous substitution of the coding region.

## Discussion

In this study, a virus was isolated from infected minks from a commercial mink farm in China. The virus, MEV/LN-10, was identified as a mink enteritis virus, based upon its cytopathic effect in F81 cells, electron-microscopical structure, physicochemical, biological and serological properties and experimental infection studies in minks.

In addition to high mutation rates and positive selection of mutations in the viral capsid gene, genetic recombination has also been determined to be an important factor in parvovirus evolution [10]. Shackelton et al. found some evidence of natural recombination among porcine, Aleutian mink disease, and several rodent parvoviruses by analyzing the genetic data deposited in databases [23]. In 2008, Mochizuki et al. provided the first clear evidence of recombination events between CPV antigenic types under field conditions. More specifically, they identified recombination between vaccine CPV-2 and field CPV-2a and CPV-2b viruses in dogs vaccinated with live combined vaccine containing CPV-2 [9]. Another research group then provided evidence of recombination events between CPV and FPLV under natural conditions [10]. In this study, recombination analysis of the MEV/LN-10 genome was detected using the GARD and RDP3 programs. The results revealed that MEV/LN-10 possessed the highest similarity to a putative parental CPV–447 in its genomic region from nucleotide 1–2,707, while, at the same time, sharing the highest sequence similarity with MEVB from nucleotide 2,708–4,269.

We analyzed the sites of key amino acids in the VP2 and NS1 proteins of the MEV/LN-10 strain and found that amino acids at positions 80, 93, 103, 323, 564 and 568 of VP2, which affect the host-range and antigenic properties of parvoviruses isolated from carnivores [24], are conserved in MEV/LN-10. The only mutations observed in MEV/LN-10 were at position 234 resulting in a change from a His to a Tyr, and three amino acids mutation at positions 247, 248 and 540 in the NS1 protein resulting in His to Gln, Thr to Ile and Val to Ala changes respectively. Of these amino acid mutations, His247Gln is also present in the recombinant strain FPLV XJ-1 [10] and other CPV.

The mutations, at position 234 of VP2 protein and 540 of NS1 protein, are new and have not been reported previously in MEV. Further work is required to determine their significance in the infection of mink by MEV/LN-10. Four mutations in VP2 at residues 87, 101, 300, and 305 have been shown previously to alter host range, transferrin receptor binding, and antigenic structure in parvoviruses [25]. For example, in a recent report by Allison et al. the authors showed that a CPV-2 mutant with only an Asp change at position 300 exhibited a 10-fold-lower infectivity for canine cells compared to feline cells [26]. Therefore the VP2 residue 300Val, seen in the mink viruses but generally not in others, may cause a loss of canine host range and alter the antigenic properties of the virus.

CPV-2 emerged as a new causative agent of severe enteritis in dogs in 1978, and it probably derived as a variant of FPLV or of a closely related virus infecting another carnivore. Subsequently, CPV-2 was replaced in nature by antigenic variants (CPV-2a, 2b, and 2c) which now coexist in dog populations worldwide [27,28]. After its emergence, CPV spread to most populations of domestic and wild carnivores [4]. We believe that CPV infection in mink occurs in nature even though CPV-2 replicates at low titers in mink following experimental inoculation [29]. Super-infection and co-infection with multiple parvovirus strains have occured, potentially facilitating recombination and high genetic heterogeneity. Indeed, Viera et al. reported co-infection with CPV variants 2b and 2c in dogs [30]. Battilani et al. also reported co-infection with CPV variants 2a and 2c in dogs and CPV and FPLV in cats [31,32]. We believe that, in the case that we have described herein, unvaccinated minks were co-infected with MEV and CPV, and the strain MEV/LN-10 was generated by a recombination event between MEV and CPV when MEV superinfected the mink already infected with CPV, or vice versa.

In a previous study, different evolutionary patterns of parvoviruses isolated from wild animals and non-domestic cats and dogs were described, highlighting the potential role of alternative hosts (e.g. raccoons) in providing indirect viral emergence pathways [26]. Mink may therefore act as an intermediate host providing parvoviruses the possibility of transmitting between divergent hosts. To clarify this further, more MEVs need to be isolated, fully sequenced and analyzed.

## Competing interests

The authors declare that they have no competing interests.

## Author contributions

JK Wang wrote the manuscript and carried out the experiments with the help of S Yang who carried out virus isolation, L Yi contributed to the recombination analysis, SP Cheng carried out sequence analysis, YN Cheng carried out PCR and HL Xu participated in the sequence alignment. XJ Yan, H Zhao and H Wu revised the manuscript. All the authors have read and approved the final manuscript.
